# Multiple sexual partnership among adolescent boys and young men in Ghana: analysis of the 2003–2014 Ghana Demographic and Health Survey

**DOI:** 10.1186/s41182-022-00484-7

**Published:** 2022-11-28

**Authors:** Isaac Yeboah, Joshua Okyere, Nutifafa Eugene Yaw Dey, Ronald Osei Mensah, Pascal Agbadi, Mary Naana Essiaw

**Affiliations:** 1 Institute of Work, Employment and Society, University of Professional Studies, Accra, Ghana; 2 Department of Population and Health, University of Cape Coast, Cape Coast, Ghana; 3 Department of Psychology, University of Ghana, P.O. Box LG 84, Legon, Ghana; 4 Centre for Languages and Liberal Studies, Takoradi Technical University, Takoradi, Ghana; 5 Department of Sociology and Social Policy, Lingnan University, SAR, Hong Kong, China

**Keywords:** Multiple sexual partnership, Adolescent boys, Young men, Social-ecological, Prevalence

## Abstract

**Background:**

Multiple sexual partnership (MSP) is a major cause of HIV/AIDS epidemic and unplanned pregnancies in sub-Saharan Africa. We investigate how individual, household, interpersonal, community and structural factors correlate with multiple sexual partnership of adolescent boys and young men in Ghana.

**Methods:**

We pooled secondary data from the 2003, 2008 and 2014 Ghana Demographic and Health Surveys (GDHS). Analytic sample of 1422 males aged 15–24 years who are sexually active and never married were used for the study. The outcome variable for the study was two or more sexual partners in the last 12 months preceding the survey. Five models were fitted using multilevel mixed effects logistic regression to identify predictors of multiple sexual partners. Results were presented using adjusted odds ratios (OR_adj_) with its corresponding 95% confidence interval.

**Results:**

The pooled data prevalence of multiple sexual partnership was 28.1%, with 18.7%, 30.0% and 33.3% of adolescent boys and young men involved in multiple sexual partnerships in 2003, 2008 and 2014, respectively. Results of the study showed that young men aged 20–24 years [OR_adj_ = 1.39, 95% CI = 1.01–1.91], being from household with richest wealth index [OR_adj_ = 1.76, 95% CI = 1.01–3.06] and those with secondary/higher education [OR_adj_ = 2.94, 95% CI = 1.44–6.06] were more likely to have multiple sexual partners. On the other hand, those who delayed their first sex [OR_adj_ = 0.45, 95% CI = 0.29–0.70] and those currently using modern contraceptive methods [OR_adj_ = 0.37, 95% CI = 0.28–0.50] were less likely to have multiple sexual partners.

**Conclusion:**

The findings provide support for the social ecological argument that sexual health behaviours are influenced by individual, interpersonal, community and contextual characteristics. Future policies and interventions seeking to address the increasing prevalence of multiple sexual partnerships among adolescent boys and young men should take into consideration family planning programmes and sexual education in affluent communities, secondary and higher institutions.

## Background

Worldwide, there has been considerable efforts in the quest to reduce and ultimately eradicate risky sexual behaviours (RSBs) among the population. Such measures adopted at the Ottawa Charter for Health Promotion (1986) and the 1995 International Conference on Population and Development (ICPD) include building healthy public policy, creating supportive environments for health, as well as meeting the sexual and reproductive health needs of individual women and men [1, 2]. Despite these policies and interventions, the incidence of HIV infections and other sexually transmitted infections (STI) remains disproportionately high among young people [3]. This high rate of STI is more profound in sub-Saharan Africa [4]. For example, global reports indicate that, about 70% of new HIV infections and 74% of HIV-related mortalities are recorded in sub-Saharan Africa [3]. This raises concerns about the possible reasons for the high incidence and mortality of STIs and HIV in sub-Saharan Africa.

Studies have linked this high rate of STIs and HIV infections in sub-Saharan Africa to involvement in risky sexual behaviours (RSBs) such as early sexual debut [5], inconsistent and non-use of condoms and intergenerational sex [6]. Beyond these factors, multiple sexual partnerships (MSPs) have been found to be associated with high risk of contracting STIs and HIV [7]. MSPs can be operationalized as having two or more sexual partners [8]. This may be concurrent or serial and doubles as a serious public health concern [9, 10]. Generally, men are more likely to report MSP behaviour than women [11]. For instance, in Malawi, 69% of young men reported having two or more sexual partners compared to 35% among females [12]. This is due to the existing gender norms and patriarchal ideologies that dominate most sub-Saharan African countries which tends to encourage and normalize the practice of men engaging in MSPs [13, 14]. Shelton [15] also posits that in most African communities, cultural norms permit polygamy and overlook male’s promiscuity on the assumption that male sexual drives cannot be controlled. This situation makes it exceedingly essential to explore the dynamics of MSPs.

Within the Ghanaian context, the phenomenon of MSPs has not been singularly investigated. The existing studies on MSPs have approached it in relation to other sexual and reproductive health issues such as its relationship with male circumcision [16] or in relation to STIs [17]. Despite these studies, few studies have been conducted to understand the prevalence and incidence of MSP and how to minimize the prevalence and incidence of MSP among adolescent boys and young men. It is important to note that the proportion of unmarried young men aged 15–24 years who had experience first sexual intercourse has more than doubled (3.6% in 2003 to 8.2% in 2014) in Ghana [18], and this has implications on young men’s number of sexual partners. Men’s number of sexual partners also have direct effect on their risk of contracting sexually transmitted diseases (STDs), including AIDS [17].

Studies have shown that individual, interpersonal, community and structural factors influence multiple sexual partnership of individual. For example, evidence from Ethiopia [10, 19], Lesotho [20] and Malawi [12] suggests that factors such as internet usage, alcohol consumption, education, occupation, age, substance abuse, religion, wealth, media exposure, age at first sexual intercourse, residence, and region are important determinants of MSPs. For example, in a study conducted by Teshale et al. [10], the authors found that alcohol consumption was associated with higher odds of MSPs. Similarly, Ahinkora et al. [16] also report that men with primary level of education and men who have been circumcised are more likely to have multiple sexual partners. Yet, little is known about the prevalence and determinants of multiple sexual partnership among adolescent boys and young men in Ghana whose sexual debut over the years had increased. This study offers insight into the prevalence of MSP within a 10-year period and factors associated with MSP. Therefore, using pooled data from three (3) different Demographic and Health Surveys, we aim to identify the determinants of multiple sexual partnership among adolescent boys and young men aged 15–24 years in Ghana. This will help to design appropriate interventions to mitigate the risk associated with multiple sexual partnership.

## Theoretical framework: social ecological approach

This study was underpinned by the social ecological model. The model explains that sexual health behaviour is influenced individual, household and interpersonal characteristics of an individual as well as institutional, community or policy factors [21]. The model assumes multiple levels of influence on an individual’s sexual behaviour. In support to this model, health behaviour is seen as an interplay of environmental and individual factors [22, 23]. This study is guided by current literature and theory, rather than by policy and macro-level structural constraints. We examine variables at the individual, household, interpersonal, community and structural levels. The individual-level variables include age, knowledge of ovulation cycle, age at first sex, education, smoking cigarettes and current contraceptive use. The household level variable used is household wealth quintile. The interpersonal level variables include exposure to family planning in formation on radio and TV. Finally, the community-level variables include religion, place of residence and ethnicity. Regarding the institutional and structural-level variables including economy, political climate, funding environment and policies, we controlled for survey year; in addition, inferences were made at the discussion of results section of this paper.

## Data

We used data from the 2003, 2008 and 2014 GDHS. The GDHS is a 5-year interval country survey, which has a representative sample of Ghanaian men and women. The survey is conducted by the Ghana Statistical Service and Ghana Health Service with technical assistance from the ICF International. The GDHS is intended to provide adequate data to monitor the population and health situation in the country [18]. The survey focuses on various aspects of demography and health by gathering data on family planning, fertility, nutrition, tuberculosis, HIV/AIDS and maternal health.

## Study participants and sample size

**Fig. 1:**
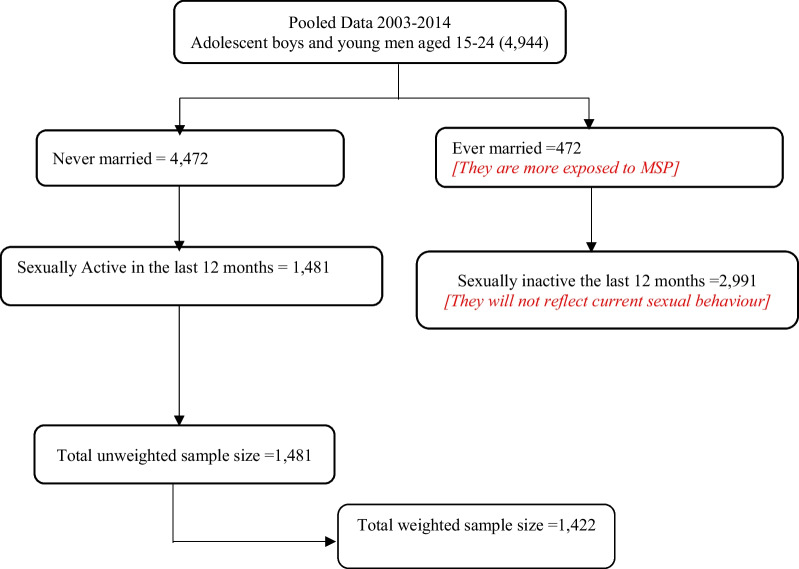
Data derived for the study

The sample for this study comprised sexually active and unmarried adolescent boys and young men aged 15–24 years. To derive a sexually active unmarried adolescent boys and young men, we first extracted the never married (4472) adolescent boys and young men from the marital status variable. Secondly, we later restricted the data to those sexually active. By sexually active, we mean adolescent boys and young men who had sexual intercourse in the last 12 months preceding the survey. This yielded a sample size of 1481. Sample weights were applied to offset the effects of under and over sampling. Univariate, bivariate and multivariate analysis of the study was conducted using a weighted sample of 1422. Permission to use the dataset was granted by Measure DHS after request for use of the dataset. Figure 1 below shows how data for analysis was derived.

## Measurement of variables

### Outcome variable

Having sex with two (2) or more partners in the last 12 months preceding the survey among sexually active unmarried young men (aged 15–24) was the outcome variable for the study. This variable was derived from the question “In total, with how many different people have you had sexual intercourse in the last 12 months?” For the purpose of this study, the variable that captured respondent’s sexual partners was recoded into “Single partner = 0” for those who reported that they had sex with only one partner and “Multiple partners = 1” representing young men who reported that they had sex with two or more partners.

### Explanatory variables

Thirteen independent variables were used. These independent variables were further categorized into four level variables; individual-level factors, household, interpersonal/network, community-level and structural-level factors. The individual-level factors include age (coded as 15–19 = 1, 20–24 = 2); age at first sex (coded as < 15 years = 1, 15–17 years = 2, 18 + years = 3); educational attainment (coded as no education = 0, primary education = 1, and secondary/higher = 2); knowledge of ovulation cycle (coded as correct knowledge = 1, wrong knowledge = 2,); current contraceptive use (coded as no method = 1, folkloric/traditionalist = 2, modern method = 3) and smokes cigarettes (no = 1, yes = 2). Household-level factor used is household wealth quintile (coded as poorest = 1, poorer = 2, middle = 3, richer = 4 and richest = 5). Interpersonal or network variable used was heard of FP on TV (No = 1, Yes = 2); heard of FP on radio (no = 1, yes = 2). Community variables include place of residence (coded as urban = 1 and rural = 2); religion (coded as Christians = 1, Muslim = 2, traditionalist/spiritualist = 3, other = 4) and ethnicity (coded as Akan = 1, non-Akan = 5). Structural variable used for the study was the year of survey (coded as 2003 = 1, 2008 = 2, 2014 = 3).

### Statistical analysis

**Fig. 2:**
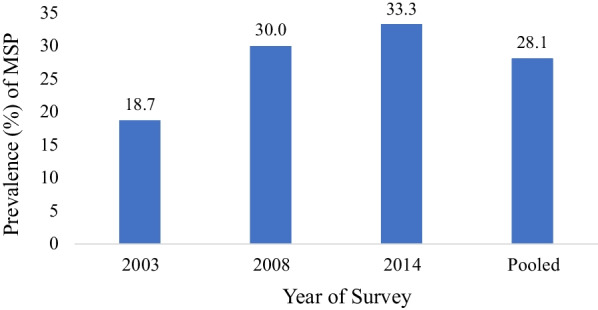
Prevalence of multiple sexual partners

All analyses were carried out with Stata version 16 (StataCorp, College Station, Texas, USA). At the descriptive level, we calculated the proportion of sexually active adolescent boys and young men (ABYM) with multiple sexual partners for each of the three surveys as illustrated in Fig. 2. Descriptive analysis was done to summarize study variables (Table 1). Variables were presented using frequencies and percentages. After this, we computed for the prevalence of multiple sexual partnerships among ABYM with respect to their socio-demographic characteristics (see Table 2). A multilevel mixed effects logistic regression which accounts for the complexity of the sampling design was used to assess the predictors of multiple sexual partnership. The mixed effects logistic regression accommodates both random and fixed effects in the model. Random effects model permit adequate control for intra cluster correlation (ICC) occasioned by the hierarchical structure of the DHS data. Five models comprising the null model (model 0) were fitted. The null model showed the variance in MSP attributed to clustering without the explanatory variables. Model I contained all individual and household level variables with year of survey as covariate. Model II contained interpersonal variables with year of survey as covariate. Model III contained community-level variables and finally, model IV contained all explanatory variables. This sequence was followed in order to understand how different levels of variables was related to the outcome. Model comparison was done using the log likelihood and Akaike’s Information Criterion (AIC) tests. The lowest AIC (1551.36) and highest log likelihood (− 751.680) were used to determine the best fit model. For all models, measure of effect was reported as adjusted odds ratio (OR_adj_) with respect to 95% confidence interval (CIs) (see Table 3). We used stata ‘xtmelogit’ command to estimate the mixed effects logistic regression models.

**Table 1:** Socio-demographic characteristics of study participants

	Frequency	Percentage
*Individual-level factors*		
Age		
15–19	500	35.2
20–24	922	64.8
Age at first sex		
< 15 years	159	11.2
15–17 years	583	41.0
18 + years	680	47.8
Educational attainment		
No education	58	4.1
Primary	217	15.3
Secondary/higher	1147	80.7
Knowledge of ovulation cycle		
Correct knowledge	363	25.5
Wrong knowledge	1059	74.5
Current contraceptive use		
No method	676	47.5
Folkloric/traditionalist	97	6.8
Modern method	648	45.6
Smokes cigarettes		
No	1396	98.2
Yes	26	1.8
*Household-level factor*		
Wealth quintile		
Poorest	191	13.4
Poorer	219	15.4
Middle	315	22.2
Richer	397	27.9
Richest	299	21.1
*Interpersonal-level factors*		
Heard FP on radio		
Yes	980	68.9
No	442	31.1
Heard FP on TV		
No	580	40.8
Yes	842	59.2
*Community-level factors*		
Religion		
Christians	949	66.7
Moslem	222	15.6
Traditional/spiritualist	149	10.5
Other	102	7.2
Ethnicity		
Akan	764	53.7
Non-Akans	658	46.3
Place of residence		
Urban	747	52.5
Rural	675	47.5
*Structural-level factor*		
Year of survey		
2003	386	27.2
2008	520	36.6
2014	515	36.2
Total	1422	100.0

**Table 2:** Prevalence of multiple sexual partnerships by selected characteristics of adolescent boys and young men between 2003 and 2014

	Number of sexual partners in the last 12 months preceding the survey		P-value
	One	Two or more	
*Individual-level factors*			
Age			
15–19	360(35.2)	141(35.2)	0.982
20–24	662(64.8)	260(64.8)	
Age at first sex			
< 15 years	99(9.7)	60(15.0)	< 0.001
15–17 years	401(39.2	183(45.6)	
18 + years	522(51.1)	158(39.4)	
Educational attainment			
No education	49(4.8)	9(2.3)	< 0.05
Primary	166(16.2)	51(12.8)	
Secondary/higher	807(79.0)	340(85.0)	
Knowledge of ovulation cycle			
Correct knowledge	277(27.1)	86(21.5)	< 0.05
Wrong knowledge	745(72.9)	314(78.5)	
Current contraceptive use			
No method	432(42.3)	244(60.8)	< 0.001
Folkloric/traditionalist	72(7.0)	26(6.5)	
Modern method	518(50.7)	131(32.7)	
Smokes cigarettes			
Yes	13(1.3)	13(3.3)	< 0.05
No	1009(98.7)	387(96.8)	
*Household-level factor*			
Wealth quintile			
Poorest	140(13.7)	50(12.5)	< 0.01
Poorer	176(17.2)	44(11.0)	
Middle	233(22.8)	82(20.5)	
Richer	276(27.0)	122(30.5)	
Richest	197(19.3)	102(25.5)	
*Interpersonal-level factors*			
Heard FP on radio			
No	301(29.5)	140(35.0)	< 0.05
Yes	721(70.5)	260(65.0)	
Heard FP on TV			
No	415(40.6)	165(41.3)	0.824
Yes	607(59.4)	235(58.8)	
*Community-level factors*			
Religion			
Christians	685(67.0)	264(66.0)	0.445
Moslems	166(16.2)	57(14.2)	
Traditionalist/spiritualist	99(9.7)	49(12.3)	
Other	72(7.0)	30(7.5)	
Ethnicity			
Akan	547(53.5)	217(54.1)	0.840
Non-Akans	475(46.5)	184(45.9)	
Place of residence			
Urban	520(50.9)	227(56.8)	< 0.05
Rural	502(49.1)	173(43.3)	
*Structural-level factor*			
Year of survey			
2003	314(30.8)	72(18.0)	< 0.001
2008	364(35.7)	156(39.0)	
2014	343(33.6)	172(43.0)

**Table 3:** Multilevel mixed effects logistic regression showing predictors of multiple sexual partnership among adolescent boys and young men between 2003 and 2014

	Model 0 (null model)	Model I	Model II	Model III	Model IV
		OR_adj_ (95% CI)	OR_adj_ (95% CI)	OR_adj_ (95% CI)	OR_adj_ (95% CI)
Fixed effects results					
*Individual-level factors*					
Age					
15–19 (ref)					
20–24		1.43(1.05–1.94)*			1.39(1.01–1.91)*
Age at first sex					
< 15 years (ref)					
15–17 years		0.82(0.54–1.24)			0.83(0.54–1.26)
18 + years		0.47(0.30–0.72)**			0.45(0.29–0.70)***
Educational attainment					
No education (ref)					
Primary			1.52(0.78–3.26)		1.77(0.84–3.73)
Secondary or higher			2.23(1.14–4.33)		2.94(1.44–6.03)*
Knowledge of ovulation cycle					
Correct (ref)					
Wrong		1.18(0.86–1.60)			1.10(0.80–1.51)
Current contraceptive use					
No method (ref)					
Folkloric/traditionalist		0.61(0.35–1.06)			0.62(0.35–1.08)
Modern method		0.39(0.29–0.52)***			0.37(0.28–0.50)***
Smokes cigarettes					
No (ref)					
Yes		1.66(0.71–3.89)			1.93(0.80–4.65)
*Household-level factor*					
Wealth quintile					
Poorest (ref)					
Poorer		0.88(0.56–1.38)			0.78(0.49–1.25)
Middle		1.02(0.67–1.56)			0.86(0.54–1.38)
Richer		1.70(1.13–2.53)*			1.35(0.82–2.23)
Richest		2.11(1.37–3.25)**			1.76(1.01–3.06)*
*Interpersonal-level factors*					
Heard FP on radio					
No (ref)					
Yes			0.86(0.65–1.18)		0.89(0.65–1.21)
Heard FP on TV					
No (ref)					
Yes			1.09(0.82–1.47)		0.96(0.70–1.33)
*Community-level factors*					
Religion					
Christians (ref)					
Muslims				0.87(0.60–1.27)	0.88(0.59–1.30)
Traditionalist/spiritualist				1.54(1.05–2.27)	1.60(1.07–2.41)
Other				1.17(0.73–1.88)	1.25(0.76–2.06)
Ethnicity					
Akan (ref)					
Non-Akans				0.85(0.65–1.12)	1.04(0.78–1.40)
Place of residence					
Urban (ref)					
Rural				0.72(0.55–0.93)*	0.98(0.69–1.38)
*Structural-level*					
Year of survey					
2003 (ref)					
2008		1.87(1.31–2.66)**	1.98(1.39–2.80)***	1.94(1.37–2.76)***	1.691(1.17–2.44)***
2014		2.28(1.59–3.28)***	2.19(1.54–3.13)***	2.19(1.54–3.12)***	1.97(1.35–2.87)***
Random effects result					
AIC	1696.482	1564.51	1604.276	1604.213	1551.36
ICC	0.049	0.067	0.077	0.074	0.071
LR test	4.83**	3.21*	4.68*	4.21*	3.15*
Log likelihood	− 845.241	− 758.123	− 794.138	− 793.106	− 751.680
Wald Chi-square	Reference	88.40***	30.36***	32.71***	97.90***
Observations	1422	1422	1422	1422	1422

*p* value < 0.05 = *, *p* value < 0.01**, *p* value < 0.001***

## Results

### Socio-demographic and contextual characteristics

Figure 2 indicates that the pooled data prevalence of multiple sexual partnership was 28.1%. It was found that MSP has increased from 18.7% in 2003 to 30.0% in 2008 and further to 33.3% in 2014.

Summary statistics was employed to describe the characteristics of the adolescent boys (15–19 years) and young men (20–24 years), which are presented in Table 1. Of the 1422 young men, 64.8% were aged 20–24 years. About 48% experienced first sex at age 18 years and above while 11% and 41% had their sexual debut when < 15 years and between 15–17 years, respectively. Most (80.7%) of the study participants had attained secondary or higher education while only 4.1% had no education. More than two-thirds (74.5%) had wrong knowledge of ovulation cycle. While close to half (47.5%) of them are not using any contraceptive method, about 46% are using modern contraceptive method.

Further, only 1.8% of the study participants smoke cigarettes. Regarding household level factor, about 28% belong to households with richer wealth quintile while a little over one-tenth (13.4%) belong to households with poorest wealth quintile. Regarding interpersonal level factors, while more than two-thirds (68.9%) reported of information of family planning (FP) information on radio, 59.2% reported of information on FP information on TV. Concerning community-level factors, two-thirds (66.7%) of the study participants are Christians, followed by Muslims who constitute 15.6%. More than half (53.7% and 52.5%) of the study participants belong to the Akan ethnic group and reside in Urban areas, respectively. Among 1422 study participants, 36.6%, 36.2% and 27.2% were ABYM who participated in the 2008, 2014 and 2003 surveys, respectively.

### Association between MSP and characteristics of study participants

Table 2 shows that a higher prevalence of MSP was recorded among those who experience first sex at 15–17 years (45.6%), those with wrong knowledge of ovulation cycle (78.5%), those not using any contraceptive method (60.8%), cigarette smokers (96.8%), those who heard FP on radio (65.0%), those with secondary/higher education (85.0%), those residing in urban centres (56.8%) and study conducted in 2008 (39.0%).

### Determinants of multiple sexual partnership

Table 3 indicates that model IV has the smallest AIC and highest log likelihood value which illustrates that this model fits the data. With Model 4, the odds of having MSP was high among young men aged 20–24 years (OR_adj_ = 1.39;95%CI = 1.01–1.91), households with richest wealth quintile (OR_adj_ = 1.76; 95%CI = 1.01–3.06), those with secondary or higher education (OR_adj_ = 2.94;95%CI = 1.44–6.03), compared with households with poorest wealth quintile and no education, respectively. On the other hand, the odds of MSP was low among those whose first sexual experience was at the age of 18 + years (OR_adj_ = 0.45;95%CI = 0.29–0.70) and using modern contraceptives (OR_adj_ = 0.37;95%CI = 0.28–0.50) compared with sexual debut before 15 years and not using any contraceptive, respectively (see Table 3).

## Discussion

This study sought to assess and identify the determinants of multiple sexual partnership among adolescent boys and young men aged 15–24 years in Ghana. The pooled data prevalence of MSP is 28.1%. We also found an increase in MSP from 18.7% in 2003 to 30.0% in 2008 and subsequently to 33.3% in 2014. These results are consistent with a previous study reporting increasing trends of multiple sexual partnership among adult women in China [24] but inconsistent with another study reporting decline of MSP among young people in South Africa [25]. Age, age at first sex, contraceptive use, and wealth quintile were identified as individual-level predictors of MSP among adolescent boys and young men in Ghana. In terms of interpersonal factors, education attainment had association with MSP. Year of survey was a consistent structural covariate of MSP in all the four models.

With individual-level factors, young men aged 20–24 years were more likely to have multiple sexual partners compared with their adolescent (15–19 years) counterparts. This contradicts finding by Kar et al. [26] who found MSP to be more common among 18–19 year-olds than in 20–24 year-olds. This was attributed to the developmental stages that teenagers (18–19 years) go through as they transition into adulthood, as well as the fact that this stage is known for experimentation and exploration [26]. Nevertheless, life transitions, exposure and immaturity may explain this finding. In Ghana, most young men of age 20–24 years are either preparing to exit tertiary institutions or have just entered the labour markets [27]. It can be reasoned that such life transitions maybe associated with less roles and responsibilities that may not serve as a healthy distraction away from having and/or keeping multiple sexual partners. Secondly, MSP behaviour among these young adults may be attributed to greater SRH literacy exposure and acceptability of having sex outside a committed relationship [28]. Lastly, it is possible that these group of young men may have “outgrown” the behaviour of committing to only one sexual partner as a result of associated cognitive changes in that sex with multiple partners is more acceptable for men at that age group [29].

Delayed age at sexual debut was also found to be a significant individual-level predictor of MSP. Young men who debuted later (i.e., 18 + years) were less likely to have multiple sexual partners. Perhaps, this group of young men out of providence may have delayed their debut because they were able to withstand peer pressure [30, 31]. Similarly, these findings are comparable to growing evidence suggesting that early sexual debut (before 18 years) is a strong predictor of MSP due in part to youthful exuberance, peer influence and experimentation [32–37].

We found that young men using modern contraceptive were less likely to engage in MSP. The evidence for the contraceptive-MSP link has been mixed. A study in Tanzania reported independent link between condom use and multiple sexual partners [38]. On the contrary, another study in Angola, reports that youths with multiple sexual partners were consistent condom users [39]. This disparity may be due to cultural background differences in relation to sexual activity among these countries and the knowledge differentials about transmission and prevention of HIV/AIDS and sexual risk behaviours between the countries. Though there are literature to support our findings, it is possible that young men using modern contraceptives may out of the fear of pregnancy refrain from engaging in MSP, whereas those not using modern contraceptive by extension with no fear may be more inclined to indulge the habit of MSP. These findings nevertheless call for a concerted effort from relevant stakeholders towards increasing sexual and reproductive health knowledge of adolescent boys and young men in Ghana. The first step toward bettering one's health is to gain a better understanding of it. Young men with knowledge and use of contraceptives have more control over their bodies and are less likely to engage in dangerous sexual behaviour (early sexual debut, sex without condoms and sex with multiple partners) [40].

We also found that household wealth quintile was significantly related with MSP. Generally, our findings suggest that belonging to the richest income households is a risk for having multiple sexual partners perhaps because young men in these households may have adequate resources to be able to cater for the health and financial needs of numerous partners [16]. This is a cause for concern for researchers and policy-makers since each extra sexual partner an individual belonging to the highest income household has, increases the danger of exposure to sexually transmitted illnesses, such as HIV and AIDS, as well as the transmission of other infections. The evidence for the wealth-MSP link has been mixed. Some researchers have reported that people belonging to wealthier households have more sexual partners [15, 16, 32, 41], others have reported the association between poorest households and greater sexual partnerships [42, 43] while others have reported no relationship between wealth and MSP [20, 35, 44, 45]. Given these contradictory findings it would be interesting for future research to examine the likely interaction effect of gender, rural–urban residency, and socioeconomic position on multiple sexual behaviour.

Education was found to be significantly associated with multiple sexual partnership (MSP). Generally, those with secondary or higher education were more likely to have multiple sexual partners compared to those with no education. This finding is consistent with other studies [46, 47] that indicated that educated adolescent boys and young men are exposed to multiple sexual partners. Findings from these studies suggest that the practice of engaging in multiple sexual partnerships is rampant among young men with higher education and it is influenced by several underlying factors such as staying alone in rented apartments, alcohol consumption and peer pressure. Plausibly, the influence of these underlying factors discourages the adoption of best sexual practices.

Regarding structural-level factors, we found that survey year was a consistent covariate of MSP. That is, being part of survey conducted in 2008 or 2014 increased the odds of MSP compared with being part of survey conducted in 2003. One reason for this phenomenon could be the nature of sexual and reproductive health (SRH) campaigns in the country during the selected years. It is possible that in both 2008 and 2014, SRH campaigns were not extensive and elaborate leading to increased rates of MSP. On the contrary, it is possible that SRH campaigns were adequate in periods prior to 2003, translating into a comparatively reduced rate as reported. This reasoning mirrors findings from a study among young men and women in Mozambique showing that campaigns that featured knowledge and discussion of MSP risk were effective against multiple sexual partnerships [48].

## Conclusion and implication

Summarily, we found an increase in the prevalence of multiple sexual partnership among adolescent boys and young men from 18.7% in 2003 to 33.3% in 2014. We also found that young men aged 20–24 years were more likely to have multiple sexual partners compared to their adolescent (15–19 years) counterparts. Additionally, adolescent boys and young men from household with richest wealth and those with secondary/higher education are more likely to have multiple sexual partners. Likewise, adolescent boys and young men who delayed their sexual debut and those currently using modern contraceptive methods were less likely to have multiple sexual partners.

Our findings suggest that programmes targeted to reduce the number of sexual partners among young people in Ghana should endeavour to design integrated interventions that address the determinants of MSP at various levels of influence. This study suggests that behaviour interventions that attempt to decrease the odds of multiple sexual behaviour by solely focusing on individual factors are unlikely to succeed over an extended period of time because the interpersonal, household and structural forces that make young men prone to multiple sexual partners are all within social norms present in the societal conditions in which people live. Furthermore, future policies and interventions seeking to address the increasing prevalence of multiple sexual partnerships among adolescent boys and young men should take into consideration family planning programmes and sexual education in affluent communities, secondary and higher institutions.

## Data Availability

The datasets generated and/or analysed during the current study are available in the MEASURE DHS database at repository; http://dhsprogram.com/data/available-datasets.cfm.

## Abbreviations

HIV: Human Immuno Virus
AIDS: Acquired immuno deficiency syndrome
GDHS: Ghana Demographic and Health Surveys
RSBs: Risky sexual behaviours
ICPD: International Conference on Population and Development
STIs: Sexually transmitted infections
SSA: Sub-Saharan Africa
MSPs: Multiple sexual partnerships
STDs: Sexually transmitted diseases
CIs: Confidence interval
ORs: Odds ratios
SRH: Sexual and reproductive health
FP: Family planning
ABYM: Adolescent boys and young men

## References

[1] Gilby L, Koivusalo M, Atkins S (2021). Global health without sexual and reproductive health and rights? Analysis of United Nations documents and country statements, 2014–2019. BMJ Glob Health.

[2] Tarkang E, Pencille L, Amu H, Komesour J, Lutala P (2019). Risky sexual behaviours among young people in sub-Saharan Africa: how can parents use the Ottawa Charter for Health Promotion for change?. SAHARA-J J Soc Aspects HIV/AIDS..

[3] Unicef Data. Global and regional trends—UNICEF DATA. 2019. Retrieved from https://data.unicef.org/topic/hivaids/global-regional-trends/.

[4] Kangmennaang J, Mkandawire P, Luginaah I (2019). Determinants of risky sexual behaviours among adolescents in Central African Republic, Eswatini and Ghana: evidence from multi-indicator cluster surveys. Afr J AIDS Res.

[5] Nelson KM, Perry NS, Stout CD, Carey MP (2020). Sexual debut among 14–17-year-old sexual minority males: a preliminary investigation of early HIV risk and sexual health needs. J Acquir Immune Defic Syndr (1999)..

[6] McCloskey LA, Eloff I, Doran K (2021). Determinants of intergenerational sexual relationships and HIV risk among South African women outpatients in Gauteng. AIDS Care.

[7] Mabaso M, Sokhela Z, Mohlabane N, Chibi B, Zuma K, Simbayi L (2018). Determinants of HIV infection among adolescent girls and young women aged 15–24 years in South Africa: a 2012 population-based national household survey. BMC Public Health.

[8] Mutinta G (2014). Multiple sexual partnerships and their underlying risk influences at the University of KwaZulu-Natal. J Hum Ecol.

[9] Althoff MD, Anderson-Smits C, Kovacs S, Salinas O, Hembling J, Schmidt N, Kissinger P (2013). Patterns and predictors of multiple sexual partnerships among newly arrived Latino migrant men. AIDS Behav.

[10] Teshale AB, Worku MG, Tesema GA (2021). Spatial distribution and factors associated with multiple sexual partnerships among reproductive-aged men in Ethiopia: a spatial and mixed-effect analysis of the 2016 ethiopian demographic and health survey. HIV/AIDS (Auckland, NZ).

[11] Onoya D, Zuma K, Zungu N, Shisana O, Mehlomakhulu V (2015). Determinants of multiple sexual partnerships in South Africa. J Public Health.

[12] Sathiyasusuman A (2015). Associated risk factors of STIs and multiple sexual relationships among youths in Malawi. PLoS ONE.

[13] Nalukwago J, Crutzen R, van den Borne B, Bukuluki PM, Bufumbo L, Burke HM, Field S, Zikusooka A, Fiedler AA, Alaii J (2019). Gender norms associated with adolescent sexual behaviours in 487 Uganda. Int Soc Sci J.

[14] Nshindano C, Maharaj P (2008). Reasons for multiple sexual partnerships: perspectives of young people in Zambia. Afr J AIDS Res.

[15] Shelton JD (2009). Why multiple sexual partners?. Lancet.

[16] Ahinkorah BO, Hagan JE, Seidu AA, Torgbenu E, Budu E, Schack T (2020). Understanding the linkages between male circumcision and multiple sexual partnership among married Ghanaian men: analysis of data from the 2014 Ghana demographic and health survey. SSM Popul Health.

[17] Agyei-Asabere CH. Multiple sexual partnerships and sexually transmitted infections (Master of Arts dissertation, University of Ghana). 2016.

[18] Ghana Statistical Services, Ghana Health Service, & ICF Macro. Ghana Demographic and Health 498 Survey Key Indicators. Accra: GSS, GHS and ICF Macro. 2015.

[19] Waktole ZD (2019). Sexual behaviors and associated factors among youths in Nekemte town, East 500 Wollega, Oromia, Ethiopia: a cross-sectional study. PLoS ONE.

[20] Mhele KE (2017). Covariates of multiple sexual partnerships among sexually active men in Lesotho. Afr J Reprod Health.

[21] Golden SD, Earp JA (2012). Social ecological approaches to individuals and their contexts: twenty years of health education and behavior health promotion interventions. Health Educ Behav.

[22] Kaufman MR, Cornish F, Zimmerman RS, Johnson BT (2014). Health behavior change models for HIV prevention and AIDS care: practical recommendations for a multi-level approach. J Acquir Immune Defic Syndr.

[23] McKee N, Manoncourt E, Yoon CS, Carnegie R (2002). Involving People, Evolving Behaviour: the UNICEF experience1. Commun Dev Soc Change Paris: UNESCO.

[24] Yingying H, Smith K, Suiming P (2011). Changes and correlates in multiple sexual partnerships among Chinese adult women–population-based surveys in 2000 and 2006. AIDS Care.

[25] Muchiri E, Odimegwu C, Banda P, Ntoimo L, Adedini S (2017). Ecological correlates of multiple sexual partnerships among adolescents and young adults in urban Cape Town: a cumulative risk factor approach. Afr J AIDS Res.

[26] Kar SK, Choudhury A, Singh AP (2015). Understanding normal development of adolescent sexuality: a bumpy ride. J Hum Reprod Sci.

[27] Adeniran A, Ishaku J, Yusuf A. Youth employment and labor market vulnerability in Ghana: aggregate trends and determinants. In West African youth challenges and opportunity pathways. Palgrave Macmillan, Cham; 2020, pp. 187–211.

[28] Vamos CA, Thompson EL, Logan RG, Griner SB, Perrin KM, Merrell LK, Daley EM (2020). Exploring 541 college students’ sexual and reproductive health literacy. J Am Coll Health.

[29] Paulsen D, Platt M, Huettel SA, Brannon EM (2011). Decision-making under risk in children, adolescents, and young adults. Front Psychol.

[30] Mlambo MG, Peltzer K, Chirinda W (2016). Predictors of multiple concurrent and multiple sexual partnerships among male and female youth aged 18–24 in South Africa. J Psychol Afr.

[31] Negeri EL (2014). Assessment of risky sexual behaviors and risk perception among youths in Western Ethiopia: the influences of family and peers: a comparative cross-sectional study. BMC Public Health.

[32] Odimegwu C, Somefun OD. Ethnicity, gender and risky sexual behaviour among 531 Nigerian youth: an alternative explanation. Reprod Health. 2017;14:1–5. 10.1186/s12978-017-0284-7 PMC5282662 28143542

[33] Yaya S, Bishwajit G (2018). Age at first sexual intercourse and multiple sexual partnerships among women in Nigeria: a cross-sectional analysis. Front Med.

[34] Zuma K, Setswe G, Ketye T, Mzolo T, Rehle T, Mbelle N (2010). Age at sexual debut: a determinant of multiple partnership among South African youth. Afr J Reprod Health.

[35] Simelane MS, Vermaak K, Zwane E, Masango S (2021). Individual and community-level factors associated with lifetime number of sexual partners among women aged 15–49 in Eswatini. PLoS ONE.

[36] Dana LM, Adinew YM, Sisay MM. Transactional sex and HIV risk among adolescent school girls in Ethiopia: mixed method study. Biomed Res Int. 2019. 10.1155/2019/4523475. 10.1155/2019/4523475 PMC6620836 31346517

[37] Hardee K, Gay J, Croce-Galis M, Afari-Dwamena NA (2014). What HIV programs work for adolescent girls?. JAIDS J Acquir Immune Defic Syndr.

[38] Exavery A, Lutambi AM, Mubyazi GM, Kweka K, Mbaruku G, Masanja H (2011). Multiple sexual partners and condom use among 10–19 year-olds in four districts in Tanzania: what do we learn?. BMC Public Health.

[39] Prata N, Vahidnia F, Fraser A (2005). Gender and relationship differences among 15–24 year-olds in Angola. Int Fam Plan Perspect.

[40] Finlay JE, Assefa N, Mwanyika- Sando M, Dessie Y, Harling G, Njau T, Chukwu A, Oduola A, Shah I, Adanu R, Bukenya J (2020). Sexual and reproductive health knowledge among adolescents in eight sites across sub-Saharan Africa. Trop Med Int Health.

[41] Fox AM (2010). The social determinants of HIV serostatus in sub-Saharan Africa: an inverse relationship between poverty and HIV?. Public Health Rep.

[42] Ahinkorah BO, Hagan JE, Seidu AA, Budu E, Armah-Ansah EK, Adu C, Ameyaw EK, Yaya S (2021). Empirical linkages between female genital mutilation and multiple sexual partnership: evidence from the 2018 Mali and 2013 Sierra Leone Demographic and Health Surveys. J Biosoc Sci.

[43] Wilson CN, Sathiyasusuman A (2015). Associated risk factors of STIs and multiple sexual relationships among youths in Malawi. PLoS ONE.

[44] Ali R, Tadele A. Risky sexual behavior across extremes of wealth in sub-Saharan Africa: a meta-analysis of demographic and health surveys. Ethiop J Health Sci. 2021;231(1):159. 10.4314/ejhs.v31i1.18. 10.4314/ejhs.v31i1.18 PMC8188115 34158763

[45] Silas J. Poverty and risky sexual behaviours: evidence from Tanzania. Working Paper 2013 No. 88. ICF International, Calverton, Maryland, USA. 2013.

[46] Osuafor GN, Okoli CE (2021). Factors associated with multiple sexual partners among first-year students in a South African university. Afr J Reprod Health.

[47] Tarkang EE (2013). Condom use and number of sexual partners among secondary school female students in an urban city of Cameroon. Rwanda J Health Sci.

[48] Figueroa ME, Kincaid DL, Hurley EA (2014). The effect of a joint communication campaign on multiple sex partners in Mozambique: the role of psychosocial/ideational factors. AIDS Care.

